# Asynchronous ripple oscillations between left and right hippocampi during slow-wave sleep

**DOI:** 10.1371/journal.pone.0171304

**Published:** 2017-02-03

**Authors:** Claudio Villalobos, Pedro E. Maldonado, José L. Valdés

**Affiliations:** 1 Programa Disciplinario de Fisiología y Biofísica, I.C.B.M., Facultad de Medicina, Universidad de Chile, Independencia, Santiago, Chile; 2 Biomedical Neuroscience Institute. Universidad de Chile, Independencia, Santiago, Chile; Nathan S Kline Institute, UNITED STATES

## Abstract

Spatial memory, among many other brain processes, shows hemispheric lateralization. Most of the published evidence suggests that the right hippocampus plays a leading role in the manipulation of spatial information. Concurrently in the hippocampus, memory consolidation during sleep periods is one of the key steps in the formation of newly acquired spatial memory traces. One of the most characteristic oscillatory patterns in the hippocampus are sharp-wave ripple (SWR) complexes. Within this complex, fast-field oscillations or ripples have been demonstrated to be instrumental in the memory consolidation process. Since these ripples are relevant for the consolidation of memory traces associated with spatial navigation, and this process appears to be lateralized, we hypothesize that ripple events between both hippocampi would exhibit different temporal dynamics. We tested this idea by using a modified "split-hyperdrive" that allows us to record simultaneous LFPs from both right and left hippocampi of Sprague-Dawley rats during sleep. We detected individual events and found that during sleep periods these ripples exhibited a different occurrence patterns between hemispheres. Most ripple events were synchronous between intra- rather than inter-hemispherical recordings, suggesting that ripples in the hippocampus are independently generated and locally propagated within a specific hemisphere. In this study, we propose the ripples’ lack of synchrony between left and right hippocampi as the putative physiological mechanism underlying lateralization of spatial memory.

## Introduction

The hippocampus has been widely associated with learning and memory, playing a significant role in navigation in both humans and rodents [1,2]. Anatomically, the hippocampus is comprised of two indistinguishable parts, located in the medial temporal lobe of each hemisphere. Left and right hippocampi are commonly considered to be functional equivalents and often synchronized. In spite of this vision, there is abundant evidence suggesting the lateralization of brain functions in humans [3–5]. Furthermore, the hippocampus seems to show significant levels of compartmentalization of functions, with several studies having identified the left hippocampus to be involved in language processing and sequential organization of choices whereas the right hippocampus predominantly involved in spatial navigation in humans [6–12]. In addition, recent investigations in rodents have revealed unexpected asymmetries between left and right hippocampi at cellular and molecular level [13–16]. All this evidence have contributed to the categorization of spatial memory as a lateralized process within the hippocampus, yet the physiological mechanisms underlying this specialization remain to be elucidated. The stabilization of recently acquired memory traces, a process known as memory consolidation, has been strongly associated with sharp-wave ripple (SWR) complexes and reactivation in the hippocampus [17–20]. The precise mechanism linking memory consolidation and ripple events, high-frequency oscillatory patterns usually present during immobility and slow wave sleep is still unclear however, some studies have suggested that these oscillations could exert their influence through spike-timed dependent plasticity [21–23], thus initiating plastic changes in structures downstream of the hippocampus. This evidence highlights the importance of ripples’ temporal occurrence within the hippocampus in the development of the memory consolidation process.

Since ripples oscillations in the hippocampus are instrumental for the consolidation of memory traces associated with spatial localization, and this process appears to be lateralized, we hypothesized that these ripple events between left and right hippocampi would exhibit different temporal dynamics. To test this hypothesis, we recorded ripple events from the dorsal CA1 region of both hippocampi simultaneously in rats during sleep and compared their intrinsic features and temporal dynamics.

## Materials and methods

### Subjects

Five Sprague-Dawley male adult rats (250–350 grs) were individually housed in Plexiglas home cages, with regular chow and water *ad libitum* and maintained in a 12:12 hours dark/light cycle. All experiments were carried out in accordance with the National Institute of Health (USA) Guide for the Care and Use of Laboratory Animals (NIH Publication No. 80–23, revised 1996) and approved by the Institutional Bio-Safety and Bio-Ethical Committee of the Faculty of Medicine, Universidad de Chile (Protocol CBA# 0597 FMUCH). All efforts were made to minimize the number of animals used and their suffering.

### Surgical procedure and implants

**Fig 1:**
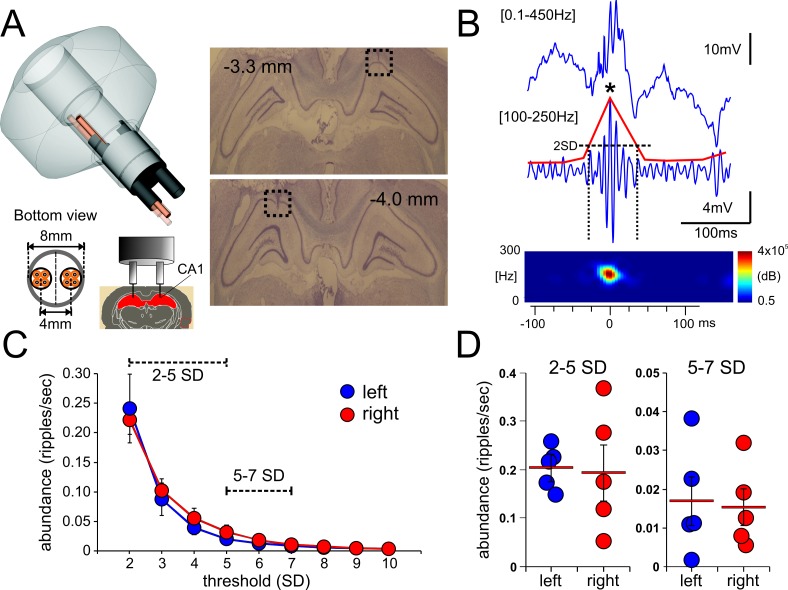
Simultaneous recording of ripple events from the left and right hippocampi. ***A*,** the left panel shows a scheme of the “split-hyperdrive” utilized for bilateral recording of ripple events. The bottom panel shows the 8-tetrode array (left) and the target location of the recording tetrodes (right). The right panel depicts two coronal slices showing the electrode track and the electrolytic-induced lesion at the tip of the recording tetrode (black dashed square) in the right (top panel, -3.3mm from bregma) and left hemisphere (bottom panel, -4.0mm from bregma). ***B***, representative broad-band recording, showing a detected ripple event (top trace). The bottom trace shows its corresponding filtered signal ([100 – 250Hz], blue trace) with the respective computed power envelope (red trace). The horizontal dotted line indicates the detection threshold while the vertical ones the time at which ripple duration was calculated. The precise time-stamp of the detected ripple event corresponds to the peak of the LFP oscillation (marked with a black asterisk). The lower panel shows the power spectrogram of the detected ripple event. ***C*,** ripple abundance throughout the recording session was not significantly different between both hippocampi regardless of the detection threshold utilized, two-way ANOVA p = 0.693. ***D*,** for a better analysis, ripple events were grouped into two groups, low amplitude (events between 2-5SD) and high amplitude (events between 5-7SD). Ripple abundance was not statistically different between left and right hippocampi when events were grouped according to their amplitude.

Rats were anesthetized with isoflurane (2.5% induction, 1.5% maintenance) in 100% oxygen at a flow rate of 1L/min. Animals were fixed in a stereotaxic frame and implanted with a custom-made “split-hyperdrive” consisting of 8 independent movable tetrodes grouped in two separated bundles (4 tetrodes per side) made of polyamide tubes (ID = 0.22mm, MicroLumen Oldsmar, FL) and positioned on each brain hemisphere (Fig 1A). Each tetrode was loaded into a silica tube (65 μm ID, 125 μm OD, Polymicro Technologies, Phoenix, AZ) which allowed tetrodes to move independently inside the polyamide tubes. A double craniotomy was performed, and the split-hyperdrive was implanted (AP: -3.2 mm from bregma; lateral: +2.2 and -2.2 from the midline; ~2.5mm depth) targeting tetrodes to the pyramidal layer of dorsal CA1 region [21]. The split-hyperdrive was then anchored to the skull with 5–6 stainless steel jewelry screws and dental acrylic. A Teflon-coated wire (stainless steel wire, 0.0045”) was welded with one of the screws in the skull and connected to the board to be used as animal ground. One of the tetrodes from the array was placed into a low activity region (corpus callosum AP: -3.2mm from bregma; lateral: +2.2 or -2.2 mm from the midline; 1.2 mm depth) and was used as reference electrode. Additionally, an EMG electrode (Teflon insulated stainless steel wire, 0.0045”) was implanted in the neck muscles, to record fine animal movement.

### Data acquisition, processing and analysis

Electrophysiological signals were simultaneously recorded from each of the 8×4 = 32 wires. Tetrodes [24] were made of four twisted 17μm nichrome wires (AM-System), gold plated to an impedance of 0.5–1 MΩ. Each tetrode was independently lowered to the dorsal hippocampus, at a speed of no more than 315 μm/day, until appropriate signals could be recorded (approximately one week until the desired region was reached). The leads of the tetrodes were connected to a unity-gain head stage, and all the data were collected using a Cheetah System (Neuralynx, Bozeman Montana). The LFP and EMG signals were acquired and digitized at 2 KHz and filtered between 0.5–450 Hz. To improve ripple event identification, selected signals were analogically filtered between 100 – 250Hz thus obtaining both broadband and ripple-specific frequency recordings. Recordings were obtained while the animal was resting on top of a flowerpot to induce spontaneous sleep and reduce movement. Each animal was recorded for at least 3 sessions, one session per day. Each recording session consisted of at least 60 minutes of continuous, uninterrupted recording while the rat was on the flowerpot. Care was taken to assure recording sessions were done at a similar time each day. In order to standardize the analysis, 50 minutes of recorded data from one of the recording session for each animal were utilized for analysis of ripple events. The identification, isolation and extraction of all the features of each ripple were completed off-line using custom-designed MATLAB routines. The best signals obtained from three separate tetrodes, two ipsi- and one contra-lateral were used for the analysis. The location of these tetrodes within each hippocampi (left or right) was completely random. Furthermore, ripple analysis demonstrated that there were no major differences in abundance nor number of coincidental events between left or right hippocampi, thus there was no bias in selecting signals from ipsi/contra-lateral tetrodes for the analysis (see Results). Broadband or analogically filtered signals were read in MATLAB, and then the mean, standard deviation (SD) and power envelope (50 ms bin) for the 50 minutes of recording were computed. Based on these values, different thresholds (mean+SD) were applied to detect ripple events. Those events whose power envelope crossed at least the 2SD threshold (Fig 1B) were detected and categorized as potential true ripple events. An extra algorithm was included within the ripple detection procedure that monitored the EMG signal in order to discard any noise produced by the movement of the animal while recording. The detected events were then grouped into two exclusive pools according to their detection threshold into low amplitude (events between 2SD and 5SD) and high amplitude (events between 5SD and 7SD). To establish relationships between ripple features and their amplitude, all ripple events were classified into either of these groups. In order to further eliminate false positive events, the individual frequency of each detected event was calculated. According to our observations, events with a frequency value below 100Hz did not fit the typical ripple profile and were discarded from the analysis. Once the events were identified as true ripples, their intrinsic features such as maximum amplitude, time of occurrence, number of cycles and frequency of the oscillation were also calculated. A graphical description of how some of these parameters were obtained is presented in Fig 1B. Once an individual event was identified, its duration was calculated as the difference between the times where the computed power envelope crossed the 2SD threshold (Fig 1B, dashed vertical lines). The amplitude of each event was calculated by measuring the highest voltage value within each event. The time of occurrence (time-stamp) for each event corresponded to the pick voltage value of each ripple event (Fig 1B, individual time-stamp for a ripple event is indicated by an asterisk). The abundance of ripples corresponds to the number of events detected in a period of time (Fig 1C). All data related to ripples detection are available on S1 and S2 Files.

### Cross-correlation parameters

Time bin width = 5 or 100 ms; window = 150 or 5000 ms; bootstrapping of the inter-ripple time = 100 or 400 shuffles, respectively. The bins at different time lags which amount of counts was 3SD higher than the bootstrapping were considered as significant cross-correlation.

### Histology

The correct location of the recording tetrodes was confirmed in all animals by electrolytic lesions (5–10μA, 10 second positive to electrode, and negative to ground) at the end of the all the recording sessions (Fig 1A). Twenty-four to forty-eight hours after the electrolytic lesion, animals were euthanized with a terminal doses of chloral hydrate 7% (1 ml/Kg) and perfused through the left ventricle with a saline flush (200 ml) followed by 250 ml of 4% paraformaldehyde in 0.1M phosphate buffer (pH 7.4). Later, the brain was carefully removed from the skull and post-fixed in the same fixative for 2 hours and then transferred to a solution of 30% sucrose in PBS (phosphate buffer 0.01M NaCl 0.9%) with 0.02% sodium azide for 2 days or until it sank to the bottom of the vial. Brains were then blocked in the coronal plane and subsequently cut with a frozen microtome into slices of a thickness of 50 μm. The tissue was then processed for Nissl staining.

### Sleep analysis

The detection and duration of the different stages of sleep was obtained by off-line analysis of the recorded EMG and LFP signals. Briefly, the root mean square (RMS) of the EMG signal was computed for the whole recording session, and then five second epochs of the EMG signal with a mean RMS value above 1SD were interpreted as the rat being in an awake state. The remained epochs were classified as either REM or SWS (slow-wave sleep) by computing the delta/theta power envelope ratio of the LFP signal in 5 seconds bins. Sleep stages were classified according to the motor activity from the EMG analysis and the predominant oscillation from the LFP signal (delta predominant = SWS; theta predominant = REM).

## Results

### Bilateral hippocampal ripple recording

In the present study we first compared different features of ripple events obtained from the dorsal CA1 region of both left and right hippocampi of rats during sleep. Sprague-Dawley male rats (n = 5) were implanted with a custom-made “split-hyperdrive” (Fig 1A). The “split-hyperdrive” configuration enabled the insertion of two bundles of 4 tetrodes into each hemisphere, allowing the recording of independent and simultaneous LFP signals. Each signal was then processed off-line to detect individual ripples (see description in methods, Fig 1B). We first compared the occurrence of ripples events between hemispheres during the recording period. As shown in Fig 1C, there were no significant differences in ripple abundance between the left and right hippocampi regardless of the detection threshold employed (n = 5; mean, SEM; two-way ANOVA, L/R x SD, p = 0.693). To compare ripple events according to their amplitude, we grouped the detected events into low and high amplitude, where low amplitude events comprised ripple events detected between 2-5SD as the threshold and high amplitude events between 5-7SD. Ripple abundance between left and right hippocampi did not show significant differences within each group (low left /right, p = 0.705, high left/right p = 0.848, t-test) independent of the event amplitude, Fig 1D. These results indicate that ripple oscillations show no statistical differences in their abundance between left and right hippocampi when recorded during sleeping periods independent of the detecting threshold.

### Ripple frequency analysis

**Fig 2:**
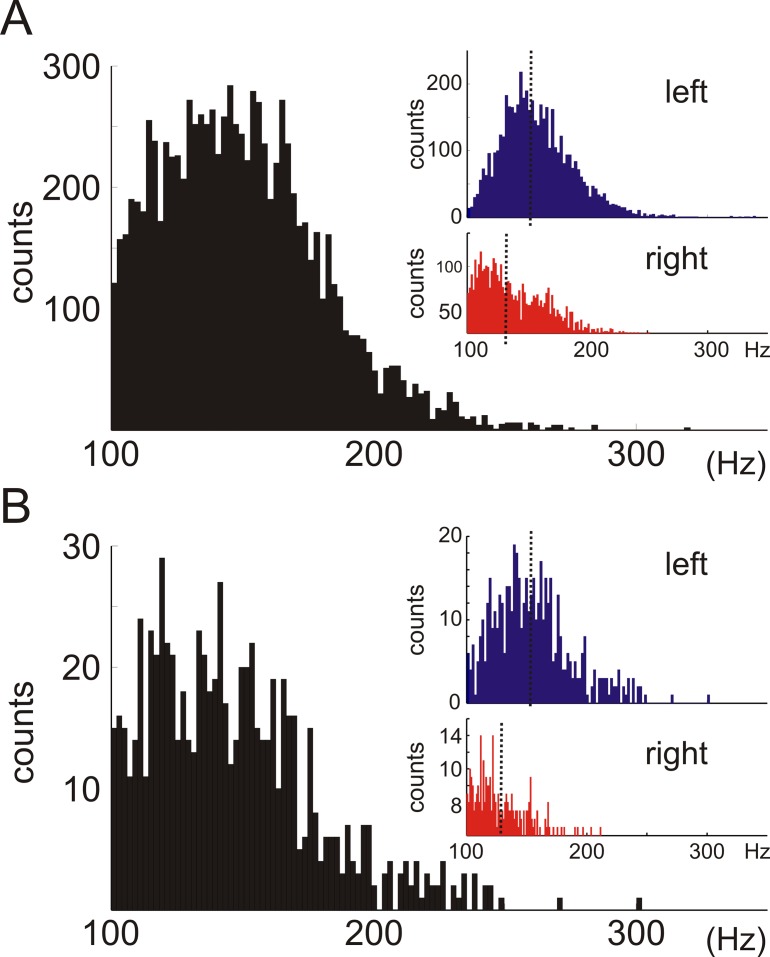
Ripple frequency analysis. Histograms depicting the distribution of the all the computed individual frequencies. Regardless of the difference in the number of events, there were no statistical differences in the mean ripple frequency values between low and high amplitude events. ***A*,** within low amplitude events, the data show differences between the mean frequency value of ripple events obtained from left (blue histogram) and right hippocampus (red histogram). ***B*,** within the high amplitude event group, the data also show statistically different mean frequency values between hemispheres.

In our recording conditions, ripple abundance did not support our hypothesis that the existence of electrophysiological differences between hippocampi could explain the lateralization phenomenon. We then evaluated whether there were any differences in the intrinsic properties of these ripple events between left and right hippocampi. We proceed to calculate the individual frequency of each of the detected ripples, assessing whether there were differences in their intrinsic frequency between hippocampi. We found significant differences in the intrinsic mean frequency value of ripples between low and high amplitude groups (p<0.001, Mann-Whitney test). Fig 2A and 2B shows histograms of all the frequency values obtained from low (upper black plot; 149.85 ± 0.32 [Hz]) and high amplitude (bottom black plot; mean freq = 146.44 ± 1.17 [Hz]), disregarding whether they came from the left or right hippocampi. When analyzing the ripple frequency from each amplitude groups now between left and right hippocampi we observed major differences in the mean frequency values of ripples obtained from the left or the right hippocampus (low amplitude, left = 158.76 ± 0.42, right = 139.75 ± 0.44 [Hz], p<0.001 Mann-Whitney test; high amplitude, left = 154.42 ± 1.42, right = 126.28 ± 1.40 [Hz], p<0.001 Mann-Whitney test). These results indicate that there were differences in the intrinsic frequency of ripple events between high and low amplitude groups (~3.41 Hz variation), but even higher differences were detected when ripples were compared between left and right hippocampi, (19.01 Hz and 28.14 Hz variation for low and high groups respectively.

### Inter-ripple interval between left and right hippocampi

**Fig 3:**
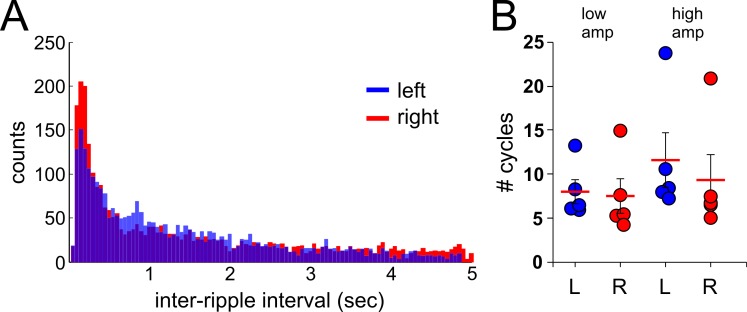
Ripple event's features between left and right hippocampi. ***A*,** inter-ripple interval (IRI) of events detected from left (blue) and right (red) hippocampi. ***B*,** mean number of cycles of ripple events between left and right hippocampi. Each circle represents the mean number of cycles for all the ripple events obtained for a recording session for a single animal. (n = 5; blue circles = left hippocampus, red circles = right hippocampus)

The differences in the mean ripple event frequency between left and right hippocampi led us to investigate whether ripples from different hemispheres showed other disparities between hemispheres. We analyzed the inter-ripple interval (IRI) between signals obtained from left and right hippocampi. Fig 3A shows the distribution of IRI values from the left and right hemispheres, with similar peak values (~150 ms) suggesting a high incidence of doublets in both hemispheres. The results presented here, showed a small but statistically difference between IRI values calculated from left and right hippocampi, respectively (mean IRI = 520.27 ± 11.32 ms; 536.03 ± 29.66 ms; p<0.001, Mann-Whitney test). We then proceed to analyze whether individual ripple events between the left and right hippocampi presented differences in the number of cycles in each oscillatory event. Fig 3B depicts the mean number of cycles for all the ripple events (low and high amplitude) from either left or right hippocampi. Our results showed no statistical differences in the number of cycles between left and right hippocampi for either low or high amplitude events. We observed no difference when individual oscillation cycles were compared between either low (left = 8.00 ± 1.38 cycles; right = 7.49 ± 1.94 cycles; p = 0.42, Mann-Whitney test) or high amplitude events (left = 11.60 ± 3.08 cycles; right = 18.679.34 ± 2.91 cycles; p = 0.15 Mann-Whitney test). These results show that ripple events between left and right hippocampi presented a similar number of cycles, a measure that can be extrapolated to event duration.

### Slow-wave sleep analysis

**Fig 4:**
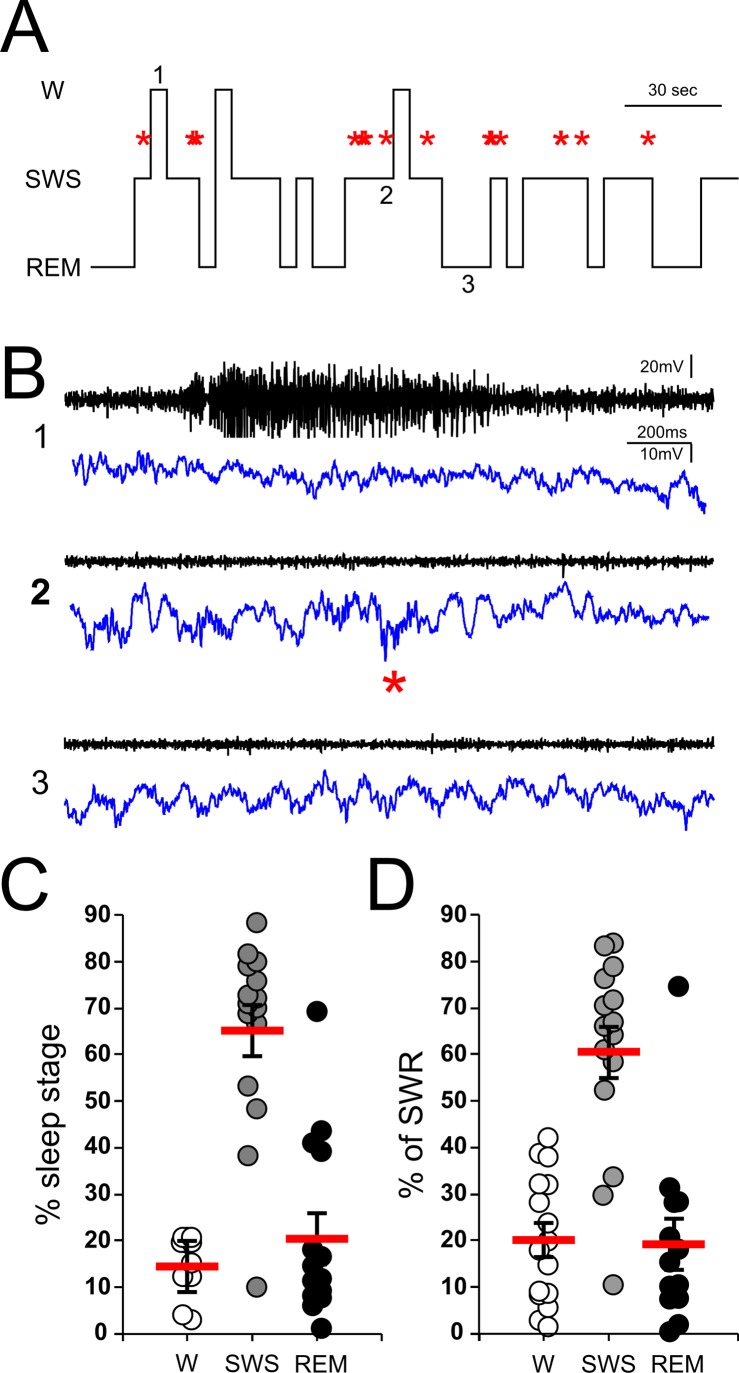
Sleep analysis. ***A*,** representative hypnogram obtained from the EMG and LFP signals showing a three-minute length single recording session depicting isolated ripple events (red asterisks) and their corresponding sleep stages (W, wake; SWS, slow-wave sleep; REM, rapid eye movement). ***B*,** EMG [100 – 450Hz] (black traces) and broadband [0.5 – 450Hz] LFP signals (blue traces) obtained during the periods marked with numbers in the hypnogram (1: wake; 2: SWS; 3: REM). A single ripple event during SWS is marked with a red asterisk. ***C*,** plot depicting the percentage of the duration of each sleep stage calculated for all the recorded LFP signals (n = 15, red line = mean, SEM). ***D*,** plot depicting the percentage of ripple events occurring on each of the wake/sleep stages (n = 15, red line = mean, SEM).

Although hippocampal ripple events were originally described as high-frequency oscillatory patterns occurring almost exclusively during slow-wave sleep (SWS), further studies have reported events in awake states and during REM sleep. Growing evidence suggest that awake and sleep ripple oscillations could serve different roles, with awake events mediating awake memory retrieval [25] whereas ripples during sleep being fundamental for memory consolidation [26]. In the present work, we focus on hippocampal-dependent memory consolidation, hence prioritizing the location and analysis of ripple events during sleep periods. In order to identify during which sleep period individual ripple events occurred sleep stages for each recording sessions were obtained by off-line analysis of the EMG and LFP signals as described in the methods section. These were classified according to the animal behavior as awake (W), slow-wave sleep (SWS) and rapid-eye movement (REM). Fig 4A and 4B represent the sleep stage analysis and the corresponding ripple events occurring during a three-minute recording session segment. Full sleep analysis revealed that animals spent the majority of the recording time in a sleep stage (W = 14.49 ± 1.48%; SWS = 65.11 ± 1.48%; REM = 20.38 ± 4.92%). Concomitant with SWS being the predominant sleep stage, ripple events occurred mostly during this sleep period (percentage of total ripple events, W = 20.21 ± 3.64%; SWS = 60.53 ± 5.44%; REM = 19.25 ± 5.00%) (Fig 4C and 4D). These results show that in our recording system, under the conditions previously described, most of the ripples detected occurred during SWS periods. We detected some spare ripple events during REM sleep most likely attributed to detection flaws during transition from SWS/REM stages (S1 Fig).

### Asynchronous ripple events between left and right hippocampi

**Fig 5:**
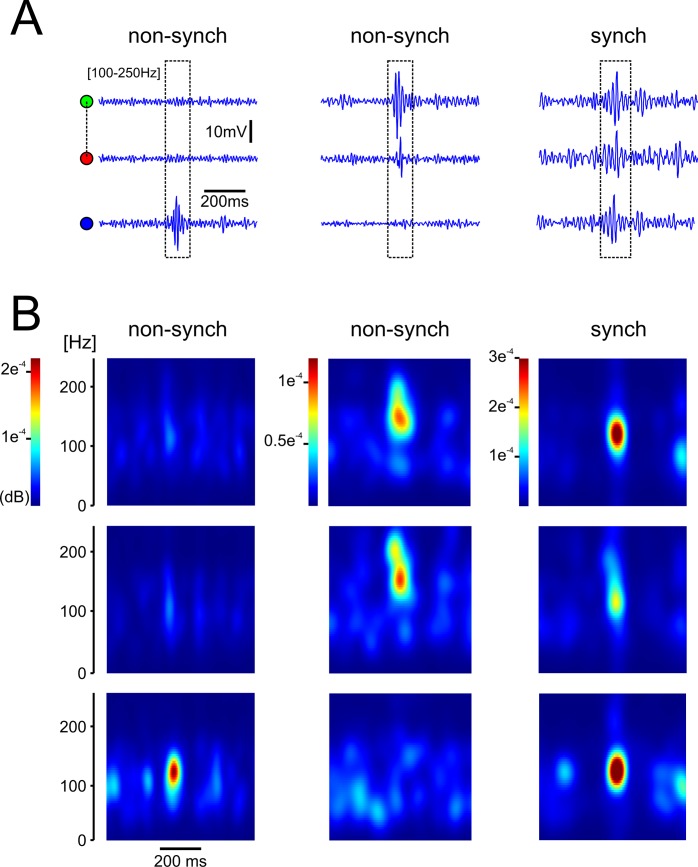
Ripple events can occur synchronously and asynchronously between left and right hippocampi. ***A*,** representative recordings of filtered LFP traces [100 – 250Hz] showing synchronous and non-synchronous ripple events between hippocampi. The left panel shows no ripple events in the signals recorded from tetrodes located in the left hippocampus (linked green and red circles) and a single ripple event in the contra-lateral right hippocampus (blue circle). The middle panel shows synchronous ripples events in the signals recorded from the left hippocampus and no events in the contra-lateral one. The right panel shows synchronous ripple events detected in both, left and right hippocampi. ***B*,** power spectrograms of ripple events between left and right hippocampi. The panels present the power spectrograms of the filtered LFP signals showed above depicting asynchronous (left and middle) and synchronous ripple events between hippocampi.

A general principle of the functional role of oscillations is that slow oscillations, characterized by relatively low intrinsic frequencies such as delta and theta waves, are generally able to travel further distances and link through their activity remote areas of the brain. On the other hand, fast oscillations such as hippocampal ripples (with a range of frequency between 100–250 Hz) are less capable of traversing large distances and thus more likely to be restricted to local circuits [27–30]. This basic principle of traveling waves supports the idea of high-frequency oscillations being spatially restricted within the hippocampus. Thus ripple oscillations could provide the physiological substrate underlying spatial memory lateralization. In agreement with this idea, Buzsaki et al., 2003 [30] described high coherence values for theta and gamma frequencies in the mouse between left and right hippocampi; however, coherence values for oscillations above 100Hz decreased rapidly perhaps indicating temporal differences between left and right hippocampi within high-frequency oscillations. To further test this idea, we compared ripple events obtained simultaneously from both hippocampi. As shown in Fig 5A, when analyzing LFP signals filtered between 100–250 Hz we found non-synchronous ripple events between contra-lateral hemispheres (left panel), synchronous events between ipsi-lateral events (center panel), and events that were coincidental in both hemispheres simultaneously (right panel). In order to verify the true nature of these ripple events and to rule out false positive artifacts, we proceeded to calculate the power spectrograms of ripple events recorded from both hippocampi. The corresponding power spectrograms (Fig 5B) confirmed the true nature of the ripple oscillations and the absence of events in contra-lateral hemispheres (left and middle panels) as well as the temporal synchrony of ripple events in both hippocampi (right panel). This figure corroborates the idea that ripple events can be found synchronously as well as asynchronously in LFP signals recorded during sleep between left and right hippocampi.

### Most ripple events are asynchronous between left and right hippocampi

**Fig 6:**
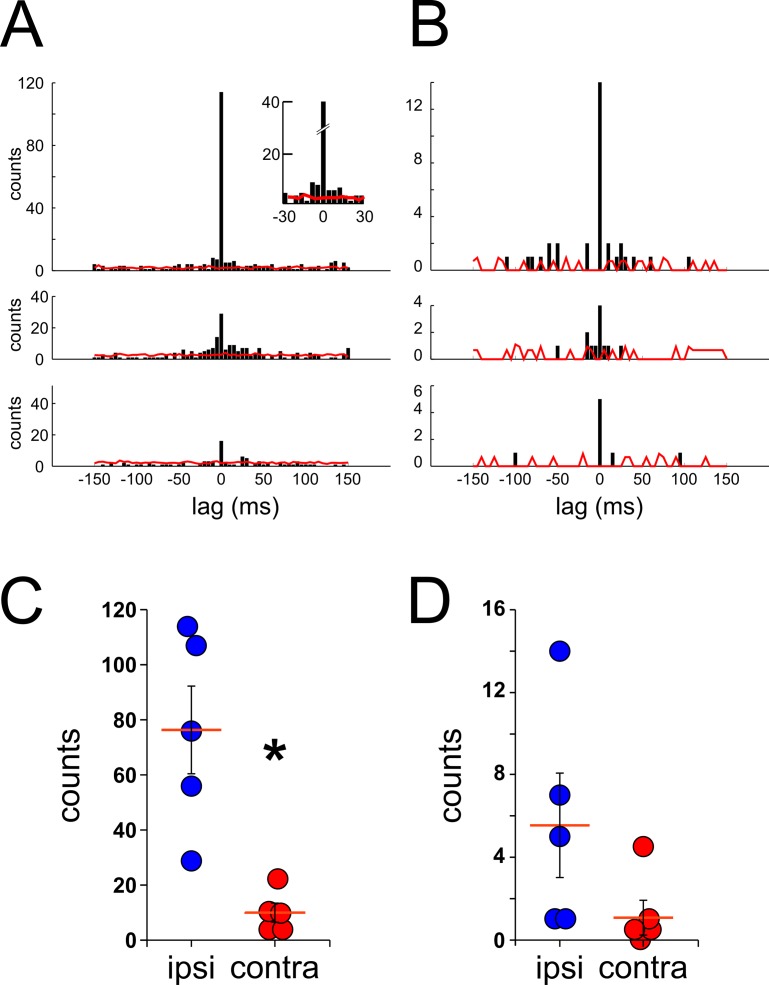
Cross-correlations of ripple events between left and right hippocampi. Cross-correlations histograms (time bin width = 5 ms) of ripple’s time-stamps between ipsi- and contra-lateral signals. ***A***, Cross-correlogram analysis of ripple events (low amplitude group) detected in a single session from tetrodes located ipsi- (upper plot) and contra-lateral (middle and bottom plot). The red trace on each histogram represents the significance level (3 standard deviations; bootstrapping of inter-ripple times, shuffles = 100) for each of the analysis (top inset panel, shows a magnification of the histogram around 0 lag time with its respective significance level). ***B***, Cross-correlogram analysis of high amplitude ripple events. The same parameters as for the low amplitude ripple cross-correlation were used for this analysis. ***C–D*,** counts of synchronous ripple events (at 0 lag time) between ipsi- (blue circles) and contra-lateral (red circles) signals. Each circle represents the averaged count of synchronous events from the cross-correlation analysis of a single recording session from an individual animal. Cross-correlation analyses were performed with ripple events from low amplitude (***C***) and high amplitude (***D***) groups, *p<0.01, Mann-Whitney test.

Are synchronous or asynchronous ripples more frequently occurring between hippocampi? One way to investigate this question is by performing cross-correlations between the time-stamps of ripple events recorded from different tetrodes. Cross-correlations analysis of all the ripple’s time-stamps from the low amplitude group between ipsi-lateral signals shows a large number of event counts at lag 0 time (Fig 6A, upper panel). Even though scanty, we also observed counts over the significance level when analysis between ripple events recorded in contra-lateral hemispheres was performed (Fig 6A, center and bottom panel). Cross-correlation analysis using the high amplitude ripple group showed similar results between ipsi- and contra-lateral events (Fig 6B). Fig 6C and 6D depict the average count of events at 0 lag from the cross-correlation analysis, of low and high amplitude groups respectively, for all the animals in the study (low amp, ipsi = 76.40 ± 15.8, contra = 10.20 ± 3.4 counts, p = 0.008, Mann-Whitney test; high amp, ipsi = 5.60 ± 2.4, contra = 1.30 ± 0.8 counts, p = 0.056, Mann-Whitney test; n = 5; mean, SEM). These results indicate that the number of synchronous events detected by cross-correlation analysis (with a rather narrow time bin width of 5 ms) is greater in signals from ipsi-lateral than contra-lateral tetrodes, especially for ripples within the low amplitude group.

**Fig 7:**
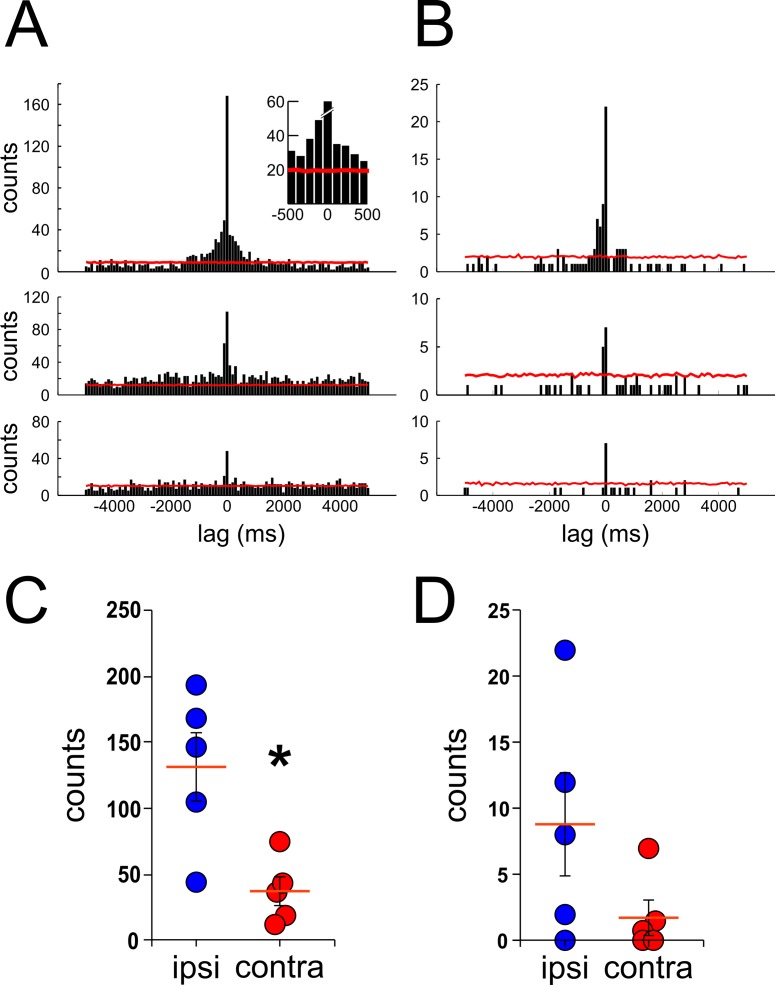
Cross-correlation analysis with a longer time bin. Similar cross-correlation analysis of ripple events as performed in Fig 6 but with a longer time bin width (100ms) for low amplitude **(A)** and high amplitude events **(B)**. The red trace represents the significance level (3SD, bootstrapping of inter-ripple times, shuffles = 400). Top panel insert depicts a magnification of the histogram around 0 lag to better show counts over the level of significance. **C–D,** counts of synchronous ripple events at 0 lag time between ipsi- (blue circles) and contra-lateral (red circles) signals. Each circle represents the averaged count of synchronous events from the cross-correlation analysis of a single recording session from an individual animal. Cross-correlation analysis was performed with ripple events from low amplitude (***C***) and high amplitude (***D***) groups, *p<0.05, Mann-Whitney test.

A previous study described that ripples can occur at any level and often travels alongside the septotemporal axis of the hippocampus [29]. These results present a challenge for the synchrony analysis since it is possible that due to the narrow time bin width utilized in the analysis a single traveling ripple can be erroneously categorized as separate asynchronous ripple events if detected with a time delay greater than the time bin width by two recording tetrodes. To avoid this, we proceed to perform cross-correlation analysis using a wider time bin width. Fig 7 shows the histograms and results of the cross-correlation analysis with a time bin width of 100ms. Similarly, as the previous results, Fig 7A shows a greater number of counts at 0 lag time in cross-correlations between ipsi-lateral recordings (top panel) than between contra-lateral ones (middle and bottom panel) for events within the low amplitude group. It is noteworthy that when a wider time bin width was used, a larger number of counts appeared over the significance level (red line), especially around the 0 lag time (insert, top panel). Comparable results between ipsi- and contra-lateral recordings were observed when the high amplitude ripple group was utilized for the analysis (Fig 7B). The quantification of the counts at 0 lag for all the animals utilized showed high similitude compared with results obtained with a narrow time bin width (Fig 6). Fig 7C and 7D depict the average count of events at 0 lag from the cross-correlation analysis, of low and high amplitude groups respectively, by using a 100ms time bin width for all the animals in the study (low amp, ipsi = 131.20 ± 26.1, contra = 37.10 ± 11.0 counts, p = 0.016, Mann-Whitney test; high amp, ipsi = 8.80 ± 3.9, contra = 1.70 ± 1.3 counts, p = 0.095, Mann-Whitney test; n = 5; mean, SEM). These results show that the differences seen in the number of synchronous events between ipsi- and contra-lateral recordings are independent of the time bin width utilized in the analysis. Furthermore, this evidence altogether supports our hypothesis of the existence of a temporal difference in the occurrence of ripple events between hippocampi.

Since all our recordings were obtained from tetrodes located ipsi- and contra-laterally from either left and right hippocampi we wanted to investigate whether there was any difference in the amount of synchronous events between ipsi-lateral tetrodes in either left or right hippocampi. In our analysis, we did not find statistically differences in the number of coincidental events recorded from ipsi-lateral tetrodes located in either the left or right hippocampi (5ms bin width: 85 ± 29, 94.5 ± 18.5 [p = 0.26, Mann-Whitney test]; 100ms bin width: 157 ± 11, 131 ± 26 [p = 0.17, Mann-Whitney test] for ipsi-lateral tetrodes located in the left and right hippocampi respectively. This result shows no intrinsic differences in the number of coincidental events between hemispheres, thus ruling out any bias when selecting recording tetrodes for ripple analysis from either left or right hippocampus.

**Fig 8:**
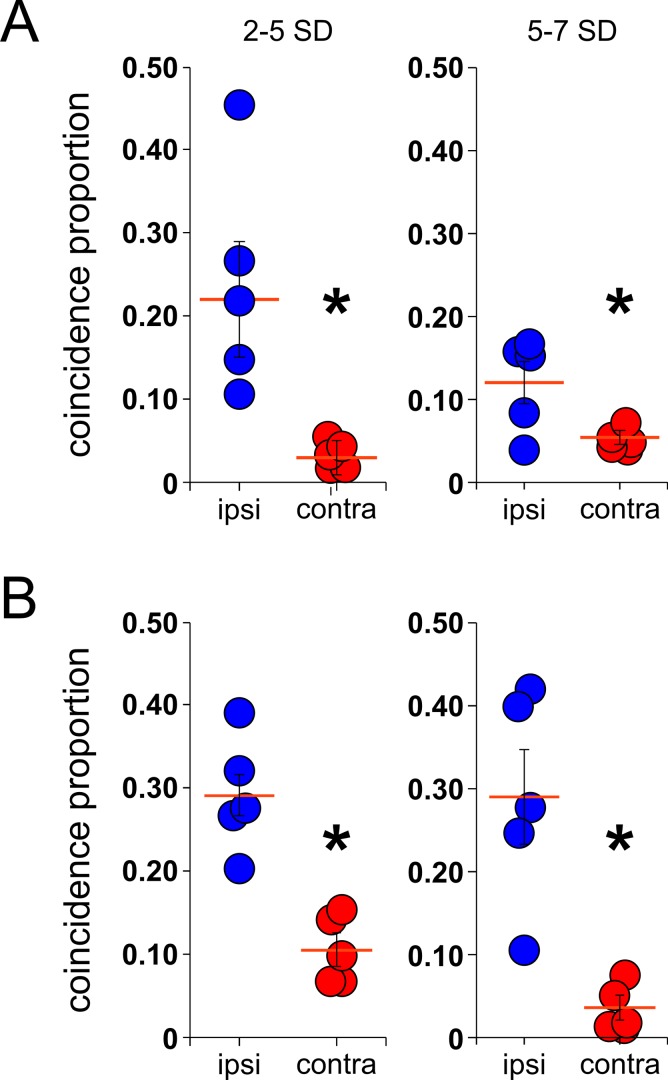
Coincidence proportion of ripple events between ipsi- and contra-lateral hippocampi. Average of coincidence proportion (number of synchronous ripples/possible events) between ipsi- (blue circles) and contra-lateral (red circles) ripple events from the low amplitude (A) and the high amplitude group (B) for all the animals recorded (n = 5, red line = mean, SEM), *p<0.01, Mann-Whitney test.

The present results suggest that the majority of the ripple events recorded during SWS are synchronous within the same hemisphere. However, a small fraction of events appeared to be bilaterally coincidental in time (Figs 6A, 6B, 7A and 7B, middle and bottom panels). Which ripple events synchronously occurred inter-hemispherically? Of the total number of ripples detected in our study (~6000) 22.4 ± 3.9% of them were classified as local events. These were defined as ripples recorded in only one of two ipsi-lateral tetrodes, thus representing spatially restricted events. Of the remaining non-local events, we wanted to investigate which proportions were classified as synchronous between ipsi- and contra-lateral recordings. A previous study has linked ripple amplitude with spatially extended patterns within the hippocampus [29], therefore to determine whether large amplitude ripples were more likely to occur inter-hemispherically, we calculated the coincidence proportion of either low or high amplitude events aiming to establish a relationship between the probability of a ripple event being coincidental inter-hemispherically with its amplitude. Fig 8A shows the mean coincidence proportion of low (left panel) and high amplitude events (right panel) between ipsi- and contra-lateral recording for each of the animals utilized in this study (low amp: ipsi = 0.22 ± 0.06; contra = 0.03 ± 0.007, p<0.01, Mann-Whitney test; high amp: ipsi = 0.12 ± 0.02; contra = 0.02 ± 0.01, p<0.01, Mann-Whitney test) with a time bin width of 5ms. Similarly, Fig 8B depicts the mean coincidence proportion of low and high amplitude events between ipsi- and contra-lateral with a time bin width of 100 ms (low amp: ipsi = 0.29 ± 0.06; contra = 0.10 ± 0.01, p<0.01, Mann-Whitney test; high amp: ipsi = 0.29 ± 0.005; contra = 0.03 ± 0.01, p<0.01, Mann-Whitney test). These results show statistical differences in the probability of two events being synchronous inter-hemispherically regardless of their amplitude.

Finally, we sought to investigate whether synchronous and asynchronous ripple events presented different intrinsic properties, such as frequency or number of cycles per event. We proceeded to select and group synchronous and asynchronous events from each recording session and analyze the features of each oscillation. Our data showed no statistical differences in the mean frequency values between synchronous or asynchronous ripple events, not even when compared between low or high amplitude events (low amp: sync = 151.4 ± 3.1, async = 142.04 ± 1.0 Hz; high amp: sync = 149.2 ± 6.4, async = 147.5 ± 3.07 Hz; p = 0.932, Kruskal-Wallis, One Way ANOVA on Rank). Similarly, there were no statistically differences in the mean number of cycles between synchronous and asynchronous ripple events in spite of the relative larger number of cycles within the high amplitude compared to the low amplitude group, mostly attributed to the larger duration of the ripple events in the former group (low amp: sync = 16.3 ± 1.4; async = 13.2 ± 0.5 cycles; high amp: sync = 23.5 ± 3.1; async = 18.8 ± 1.7 cycles; p = 0.285, Kruskal-Wallis, One Way ANOVA on Rank).

## Discussion

In this study, we found for the first time that contrary to the common assumption of brain oscillations being completely synchronous between hemispheres, there are temporal differences in specific high-frequency oscillatory patterns between the left and right hippocampi. Our results show that a much greater number of ripple events recorded from the dorsal CA1 region during sleep were synchronized only between ipsi-lateral as compared to contra-lateral recordings. These findings suggest that the origin of the majority of ripple events within the hippocampus may be restricted within each hemisphere. Furthermore, the lack of synchronization of these events also suggests the existence of multiple rather than a common driver coordinating the appearance of individual ripple events, with anatomically separated inputs driving these oscillations. If ripple events were trigger by a common driver in both hemispheres, we would have expected a larger number of synchronized events recorded from contra-lateral tetrodes. Our results indicate that this is not the case, even when a wider time window (100ms) was used to analyze event synchrony in order to account for traveling ripples as well as for the temporal differences of local events due to the delays in the cell assemblies recruited for ripple initiation. The identity of the structures/sources triggering ripple events is currently unknown, and further research is needed in order to determine its functionality and location. It is noteworthy to indicate that for this study we detected and analyzed only the fast oscillatory patterns of the LFP (100 – 250Hz) within the SWR complex commonly found in the hippocampus CA1 region. A recent report [31,20] showed that CA1 ripples originated from CA2 region can be found separated from their sharp-wave component, therefore, the conclusions extracted from this study cannot be extrapolated to the sharp-wave component of the SWR complex. Further analysis and experiments should be carried out in order to establish a sharp-wave/ripple synchrony between hemispheres.

A previous study had reported a clear relation between ripple amplitude and the distance at which these events could be found within the septotemporal axis of the hippocampus [29]. Although these results were obtained unilaterally, an alternative explanation for our results could be that synchrony patters between ripple events inter-hemispherically could reflect merely the distance between contra-lateral recording tetrodes located in separated hemispheres. In our study, the maximum distance between contra-lateral tetrodes in different hemispheres did not exceed 4.5mm. Patel et al. reported that within that distance, the percentage of synchronous inter-hemispherical events calculated with a long time bin width (± 50ms) reached values close to 50%. If the whole hippocampus presented such high electrical connectivity between hemispheres that it could act as a single electrically continued entity, we should expect similar proportions of synchrony events between left and right hippocampi. Our results did not support the idea of electrical continuity between hippocampi since the proportion of synchronous events detected between hippocampi was far lower than the calculated inter-hemispherically, in spite of the distance of the recording tetrodes inter-hemispherically. Furthermore, the same study clearly demonstrated that high amplitude events are more likely to be synchronous between larger distances than low amplitude events, an opposite result to what we present in this study (Fig 8), where the proportion of synchronous low amplitude event was slightly higher than the high amplitude ones between hemispheres. All these evidence allowed us to challenge the common view of the hippocampus being a highly interconnected unit where high frequency oscillations travel unopposed between hemispheres. We, therefore, confront the idea of left and right hippocampi acting as a single interconnected entity where the spreading of high-frequency oscillations, such as ripple events, is merely dictated by the distance between their origins.

The results presented here provide the first piece of evidence supporting a physiological role of ripples in the lateralization of spatial memory in the hippocampus. However, this effect by itself does not account for the lateralization of spatial memory nor differences in memory consolidation. Previous studies have shown that the disruption of ripple events reduced the performance of a rat in a spatial task and decreased learning [17–19]. These studies highlight the importance of ripple abundance in the process of memory consolidation. In our study, we did not find differences in the abundance of ripple events between left and right hippocampi. One possible explanation resides in that our recording condition did not involve the learning of a particular task but only sleeping periods, thus making it hard to observe variations in ripple abundance linked to memory consolidation in our experiment set up. Further studies recording bilateral ripple events during sleeping periods before and after the completion of a spatial memory task will be needed to observe potential differences in ripple abundance between left and right hippocampi during spatial memory consolidation. In spite of these results, ripple frequencies as well as the inter-ripple interval, showed marked differences between left and right hippocampi, suggesting the existence of differences in the intrinsic features of ripple events between hippocampi, thus supporting the hypothesis of ripple events being important for the lateralization of memory consolidation in the hippocampus.

## Supporting information

S1 Fig Ripple at REM-SWS transition. **A,** representative traces of recordings from a single animal depicting ripple events isolated at different threshold detections. Upper panel, broad-band LFP recording. Middle panel, (100–250 Hz) filtered trace showing true (green asterisks) and false positive (red asterisks) ripple events. False positives were categorized as such if the event did not reached the mean + 2SD threshold or presented an event frequency <100Hz. Lower panel, power envCaptionelope of the filtered trace with the corresponding detection threshold levels. Mean + 1, 2 and 3 SD (red, green and black dotted lines respectively). **B,** representative recordings of a ripple event recording during REM sleep. Upper panel, EMG and broad-band LFP recording (black and blue traces, respectively) showing a real ripple (green asterisk) and a false positive event (red asterisk) detected during REM sleep. Middle panel, blue trace shows a filtered LFP signal (100–250 Hz). Lower panel, 15 second segment of the hypnogram from the corresponding recording session indicating the time where the ripple occurred (green asterisk).
(TIF)

S1 File Ripple count. File containing ripple count trough all animals and sessions.
(ODS)

S2 File Inter ripple interval and numbers of cycles. File cointaining the inter ripple interval and numbers of cycles per ripples through all animals and sessions.
(ODS)
