# Supplementary figures and images for: Asynchronous ripple oscillations between left and right hippocampi during slow-wave sleep
Source: PLoS One. 2017 Feb 3;12(2):e0171304. doi: 10.1371/journal.pone.0171304 (PMC5291648; doi:10.1371/journal.pone.0171304)

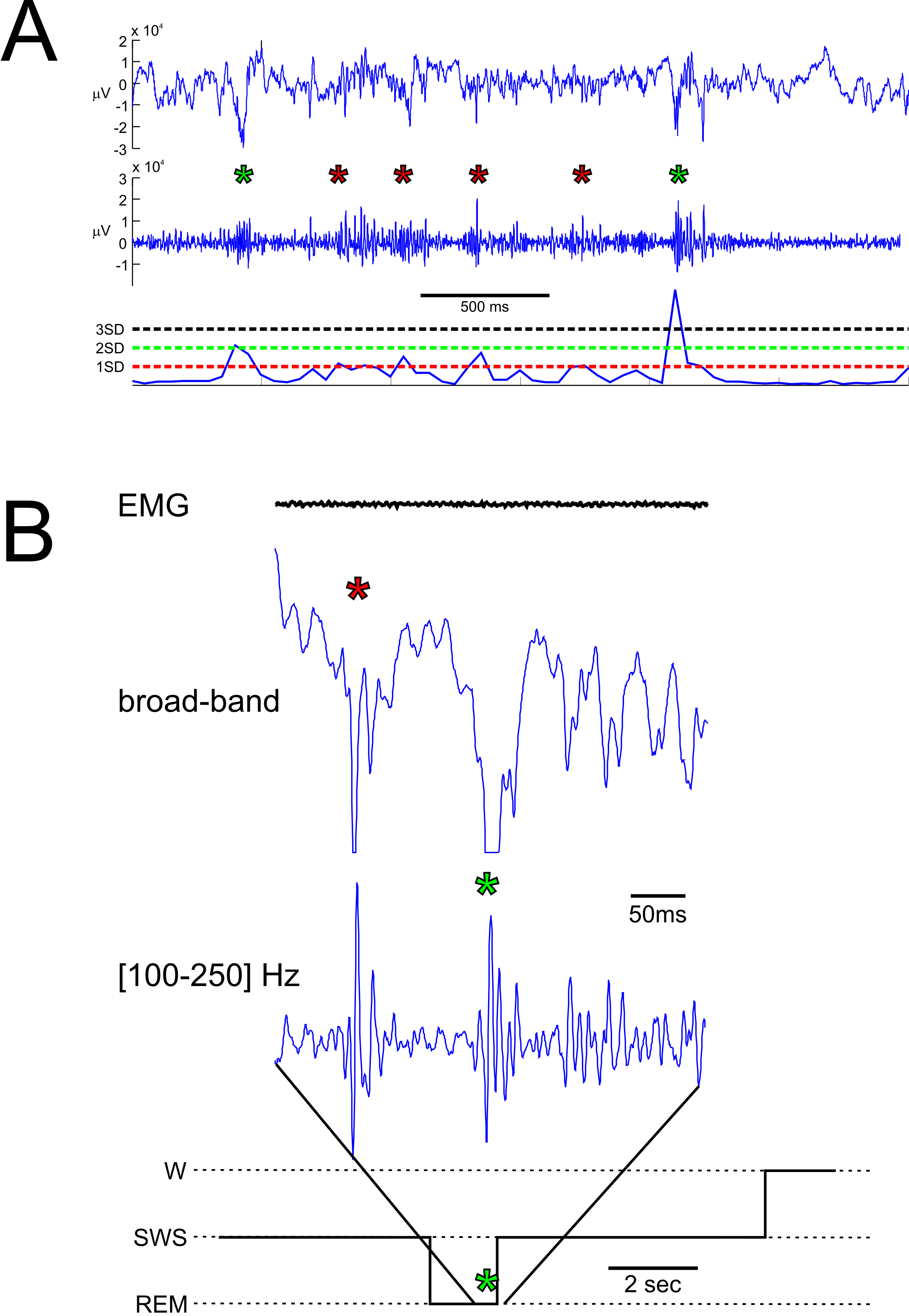

Supplement: S1 Fig — A, representative traces of recordings from a single animal depicting ripple events isolated at different threshold detections. Upper panel, broad-band LFP recording. Middle panel, (100–250 Hz) filtered trace showing true (green asterisks) and false positive (red asterisks) ripple events. False positives were categorized as such if the event did not reached the mean + 2SD threshold or presented an event frequency <100Hz. Lower panel, power envCaptionelope of the filtered trace with the corresponding detection threshold levels. Mean + 1, 2 and 3 SD (red, green and black dotted lines respectively). B, representative recordings of a ripple event recording during REM sleep. Upper panel, EMG and broad-band LFP recording (black and blue traces, respectively) showing a real ripple (green asterisk) and a false positive event (red asterisk) detected during REM sleep. Middle panel, blue trace shows a filtered LFP signal (100–250 Hz). Lower panel, 15 second segment of the hypnogram from the corresponding recording session indicating the time where the ripple occurred (green asterisk). (TIF) [file pone.0171304.s001.tif]
